# EGFR mutation testing and treatment decisions in patients progressing on first- or second-generation epidermal growth factor receptor tyrosine kinase inhibitors

**DOI:** 10.1186/s12885-020-06826-0

**Published:** 2020-04-28

**Authors:** Anne C. Chiang, Ancilla W. Fernandes, Melissa Pavilack, Jennifer W. Wu, François Laliberté, Mei Sheng Duh, Nabil Chehab, Janakiraman Subramanian

**Affiliations:** 1 Yale School of Medicine, 20 York Street, New Haven, CT 06510 USA; 2 AstraZeneca US, 950 Wind River Ln, Gaithersburg, MD 20878 USA; 3 Groupe d’analyse, Ltée, Deloitte Tower, 1190 Avenue des Canadiens-de-Montréal Suite 1500, Montreal, QC H3B 0G7 Canada; 4 Analysis Group, Inc., 111 Huntington Avenue, 14th Floor, Boston, MA 02199 USA; 5 Saint Luke’s Cancer Institute, 4321 Washington St, Medical Plaza III, Ste 4000, Kansas City, MO 64111 USA

**Keywords:** NSCLC, EGFR-TKI, Real-world, EGFR mutation testing, Treatment patterns

## Abstract

**Background:**

The objective of this study was to investigate real-world EGFR mutation testing in patients with metastatic non-small cell lung cancer (NSCLC) upon progression on first−/second-generation epidermal growth factor receptor (EGFR)-tyrosine kinase inhibitors (TKI), and subsequent treatments received.

**Methods:**

Flatiron Health electronic health records-derived database was used to identify adult patients with metastatic NSCLC treated with first−/second-generation EGFR-TKI from 11/2015–09/2017, with start of first EGFR-TKI defined as the index date. Patients were stratified by receipt of EGFR-TKI as first-line (1 L) or later-line (2 L+) treatment. Mutation testing and subsequent therapies following first−/second-generation EGFR-TKI were described.

**Results:**

Overall, 782 patients (1 L = 435; 2 L+ =347) were included. Median age was 69.0 years, 63.6% were female, 56.3% were white, 87.1% were treated in community-based practices, and 30.1% of patients died during the study period; median follow-up was 309.0 days. Among the 294 (1 L = 160; 2L+ =134) patients who received subsequent therapies, treatments included chemotherapy only (1 L = 15.6%; 2L+ =21.6%), immunotherapy only (1 L = 13.8%; 2 L+ =41.0%), and targeted therapies (1 L = 70.0%; 2 L+ =36.6%). Specifically, 40 (25.0%) 1 L patients and 7 (5.2%) 2 L+ patients received osimertinib as subsequent therapy. Before the start of subsequent therapy, EGFR T790M resistance mutation testing was performed in 88 (29.9%) patients (1 L = 63 [39.4%]; 2 L+ =25 [18.7%]). Of these patients, 25 (28.4%) were T790M positive, among whom 24 (96.0%) received osimertinib.

**Conclusions:**

A third of patients received subsequent therapies on disease progression; only 30% of these were tested for EGFR-TKI resistance mutation, prior to receiving subsequent therapies. These results highlight the importance of choosing treatments in the 1 L setting that optimize benefits for patients with EGFR-mutated NSCLC.

## Background

Systemic therapy for metastatic non-small cell lung cancer (NSCLC) varies according to tumor histology and mutation status of patients. Epidermal growth factor receptor (EGFR) mutation-positive tumors are highly responsive to EGFR-tyrosine kinase inhibitor (TKI) therapy [1–5]. EGFR-TKIs provide improved clinical benefits, quality of life, and are considered more tolerable than chemotherapy [1–5]. Clinical guidelines recommend routine mutation testing and identification of EGFR mutations in all patients with NSCLC of non-squamous cell carcinoma (non-SCC) histology to identify who may benefit from approved EGFR-TKIs [6–8]. However, most patients develop resistance to first- or second-generation EGFR-TKIs [9], with T790M, a resistance mutation in the EGFR gene, observed in approximately 50% of these patients [10, 11]. EGFR T790M results in reduced binding of first- or second-generation EGFR-TKIs, lessening EGFR-TKI-mediated inhibition and leading to disease progression [12, 13]. Identification of T790M prompted development of different therapeutic strategies to overcome resistance to EGFR-TKIs.

Osimertinib, a third-generation irreversible EGFR-TKI, selectively inhibits both EGFR-TKI sensitizing mutations and T790M. In November 2015, osimertinib received accelerated approval by the US Food and Drug Administration (FDA) to treat patients with EGFR T790M positive NSCLC whose disease progresses on or after prior treatment with EGFR-TKIs [14]. In April 2018, the FDA approved osimertinib as a first-line (1 L) treatment option for patients with EGFR exon 19 deletion (ex19del)/L858R-positive metastatic NSCLC [15]. Furthermore, final overall survival results from the FLAURA study demonstrated significantly longer overall survival in those who received osimertinib versus comparator EGFR-TKI, with a 20% lower risk of death among patients with untreated advanced NSCLC with an EGFR-TKI sensitizing mutation [16]. Therefore, improved understanding of how EGFR mutation testing and treatment patterns have evolved in real-world practice among patients with metastatic NSCLC treated with first- or second-generation EGFR-TKIs, since approval of osimertinib, is needed. Clinical guidelines recommend T790M testing for patients with resistance to first- or second-generation EGFR-TKIs, yet uptake of such testing in real-world practice is unknown [17]. In this real-world study, we aimed to describe the utilization of EGFR mutation testing of patients on progression with first- or second-generation EGFR-TKIs, subsequent treatments received, and the proportion of T790M positive patients treated with osimertinib.

## Methods

### Data source

This study used data from January 1, 2011 to September 30, 2017 from the Flatiron Health database, which is derived from electronic health records (EHR) containing longitudinal, patient-level data from a demographically and geographically diverse, nationally representative population in the US [18]. Additional data modules, including data on progression, site of metastases, and histology, were acquired for patients with metastatic NSCLC who received subsequent therapy after treatment with first- or second-generation EGFR-TKI to confirm disease progression. The data are de-identified, with provisions in place to prevent re-identification to protect patient confidentiality, in accordance with the 1996 Health Information Portability and Accountability Act.

### Study design and study population

A retrospective longitudinal cohort study was conducted among adult patients with metastatic NSCLC treated with a first- or second-generation EGFR-TKI. To evaluate EGFR mutation testing and treatment patterns after first FDA approval of osimertinib (November 2015), patients were selected based on use of EGFR-TKI on or after November 1, 2015. Patients included had a diagnosis of lung cancer (International Classification of Diseases, 9th Revision, Clinical Modification diagnosis code: 162.x; International Classification of Diseases, 10th Revision, Clinical Modification diagnosis code: C34x, C39.9), at least two clinical visits on or after January 1, 2011, pathology consistent with NSCLC, and a diagnosis of stage IIIB–IV NSCLC or early-stage NSCLC and subsequent development of recurrent or progressive disease on or after January 1, 2011. Additionally, patients had to be aged 18 years or more at the time of their diagnosis, treated with a first- or second-generation EGFR-TKI (erlotinib, afatinib, or gefitinib) on or after November 1, 2015, have had at least one clinical visit within 3 months prior to EGFR-TKI initiation, and have initiated a first- or second-generation EGFR-TKI at least 6 months prior to end of data (September 30, 2017) to avoid right censoring. Patients with simultaneous use of multiple EGFR-TKI therapies were excluded. The index date was the date of initiation of EGFR-TKI treatment. The observation period extended from index date to study end date (date of death, end of clinical activity, or data cutoff).

### Study measures

Demographic and clinical characteristic data collected included age at metastatic NSCLC diagnosis, sex, race, geographical region, physician practice type, insurance type, history of smoking, line of therapy for first- or second-generation EGFR-TKI, time from diagnosis (date of first recurrence or progressive disease after initial NSCLC diagnosis) to those who initiated EGFR-TKI in 1 L or second or later line (2 L+), and Eastern Cooperative Oncology Group (ECOG) performance status. Treatment patterns were assessed and included subsequent therapies following first- or second-generation EGFR-TKI. NSCLC therapies were classified according to chemotherapies, immunotherapies, and targeted therapies (Table 1). Specifically, subsequent therapy was defined as a change in treatment after receiving a first- or second-generation EGFR-TKI. Patients who received the same EGFR-TKI agent in their index and subsequent therapy were considered to have continued on their initial EGFR-TKI therapy as subsequent therapy. Patients could have been classified into multiple therapy groups if receiving combination therapy (Table 2). Data on rates and results of EGFR mutation testing were reported for two time periods: 1) from metastatic NSCLC diagnosis to index date, 2) from after index date to date of initiation of subsequent therapy. For patients with multiple EGFR mutation tests, the test closest to index date was chosen for the baseline period, and the test closest to initiation of subsequent therapy was chosen for the observation period. In the database, EGFR and T790M were tested and reported together. Different types of mutations were also reported (i.e. T790M, ex19del, L858R point mutation in exon 2, other mutation types) for patients who were tested for EGFR and had positive results.

**Table 1:** Chemotherapies, immunotherapies, and targeted therapies for metastatic NSCLC

Type of Therapy	Agents/Agent combinations
**Traditional Chemotherapies**	
Platinum monotherapy	Carboplatin, Cisplatin
Platinum doublet therapy	
With cisplatin	Docetaxel, Etoposide, Gemcitabine, Irinotecan, Paclitaxel, Pemetrexed, Vinorelbine
With carboplatin	Docetaxel, Gemcitabine, Paclitaxel, Pemetrexed, Vinorelbine
Non-platinum based combination therapies	Gemcitabine/Docetaxel, Gemcitabine/Paclitaxel, Gemcitabine/Vinorelbine, Paclitaxel/Vinorelbine, Pemetrexed/Gemcitabine
Maintenance therapy	Docetaxel, Gemcitabine, Pemetrexed
**Immunotherapies**	Atezolizumab, Nivolumab, Pembrolizumab
**Targeted Therapies**	
ALK inhibitors	Alectinib, Brigatinib, Ceritinib, Crizotinib
Angiogenesis inhibitors	Bevacizumab, Ramucirumab
BRAF inhibitor	Vemurafenib
CDK4/6 inhibitor	Palbociclib
EGFR monoclonal antibody inhibitor	Cetuximab, Necitumumab
EGFR tyrosine kinase inhibitors	Afatinib, Erlotinib, Gefitinib, Osimertinib
MEK inhibitor	Trametinib
Other monoclonal antibody inhibitors	Ipilimumab, Trastuzumab

*ALK* anaplastic lymphoma kinase, *BRAF* Raf isoform B, *CDK* cyclin dependent kinase, *EGFR* epidermal growth factor receptor, *MEK* mitogen activated protein kinase kinase

**Table 2:** Treatment combinations among patients with a subsequent line of therapy

Subsequent line of therapy	Patients who received 1 L EGFR-TKI and had subsequent therapy (***N*** = 160), *n* (%)	Patients who received 2 L+ EGFR-TKI and had subsequent therapy (***N*** = 134), *n* (%)
Chemotherapy alone	25 (15.6)	29 (21.6)
Immunotherapy alone	22 (13.8)	55 (41.0)
Targeted therapies		
EGFR-TKI		
Alone	93 (58.1)	23 (17.2)
Plus chemotherapy	3 (1.9)	3 (2.2)
Plus immunotherapy	3 (1.9)	2 (1.5)
Plus other targeted therapies	2 (1.3)	2 (1.5)
Plus chemotherapy, other targeted therapies	1 (0.6)	3 (2.2)
Other targeted therapies		
Alone	4 (2.5)	2 (1.5)
Plus chemotherapy	6 (3.8)	14 (10.4)
Clinical study drug^a^	1 (0.6)	1 (0.7)

*1 L* first line, *2 L+* second or later line, *EGFR* epidermal growth factor receptor, *EGFR-TKI* epidermal growth factor receptor-tyrosine kinase inhibitor

^a^ Flatiron Health masks the names of clinical study drugs in the data due to the sensitive nature of patients in clinical trials

Patients with subsequent therapy following EGFR-TKI treatment were identified, and additional data abstraction was conducted to identify disease progression. Clinician documentation in medical charts, radiographic assessment, and/or pathology reports from the progression module in the database were reviewed to confirm whether disease progression occurred after EGFR-TKI initiation. Death of patients was determined from EHR and two external sources, including Social Security Death Index, and a commercial death dataset which collects information from obituaries, funeral homes and other sources to provide date of death within a week of death [19]. Patients without any clinical activity 3 months before data cutoff (i.e. July 1, 2017 to September 30, 2017) were considered lost to follow-up. Patients still treated with first EGFR-TKI therapy in the month prior to data cutoff were considered remaining on therapy. Patients not treated with first EGFR-TKI therapy in the month prior to data cutoff, but with evidence of clinical activity during the 3 months prior to data cutoff, were considered as having discontinued therapy.

### Statistical analyses

Patient characteristics were described overall and separately for patients who received EGFR-TKI in the 1 L and 2 L+. Means (standard deviations) and medians were reported for continuous variables, and frequencies and proportions were reported for categorical variables. For patients who received EGFR-TKI in 1 L versus 2 L+, results were compared using Wilcoxon rank sum test for continuous variables and chi-squares tests for categorical variables. The frequency and proportion of patients with EGFR mutation testing and specific results of testing from metastatic NSCLC diagnosis to index date were reported for the overall study population. Results of EGFR mutation testing between index date and subsequent therapy were reported for those who received subsequent therapy. The frequency and proportion of patients treated with specific types of subsequent therapies were described. The frequency and proportion of patients tested for EGFR (including T790M), testing results, and specific subsequent therapies received, were reported.

## Results

### Patient characteristics

**Fig. 1:**
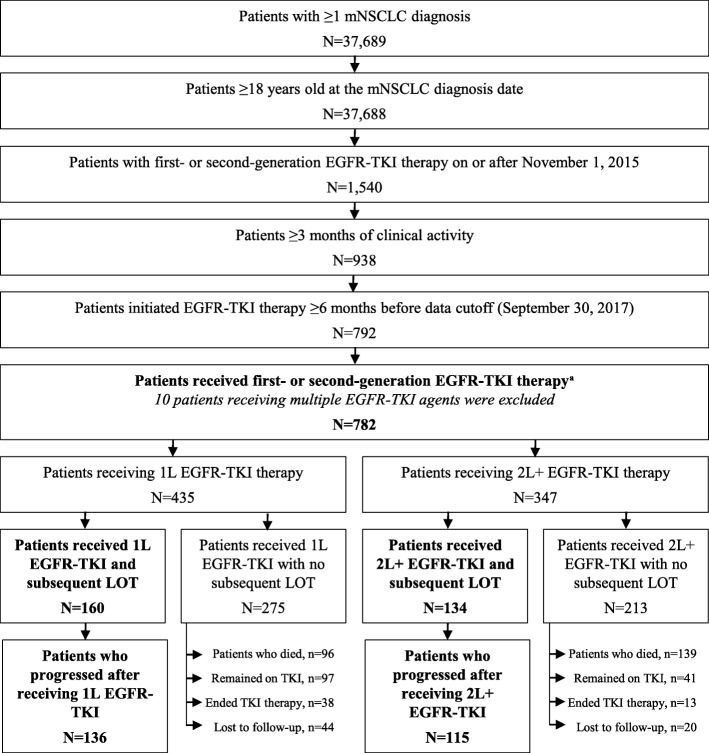
Patient selection based on study inclusion/exclusion criteria. EGFR: epidermal growth factor receptor; LOT: line of therapy; NSCLC: non-small cell lung cancer; TKI: tyrosine kinase inhibitor. ^a^ Patients receiving multiple EGFR-TKIs in the same line of therapy on or after November 1, 2015 were excluded. This includes patients who started osimertinib plus a first- or second-generation EGFR-TKI or ≥ 2 first- or second-generation EGFR-TKIs.

The overall study population included 782 eligible patients with metastatic NSCLC using a first- or second-generation EGFR-TKI (shown in Fig. 1). In total, 435 patients received an EGFR-TKI as initial systemic therapy (1 L) and 347 as subsequent therapy (2 L+). Of 435 patients who received EGFR-TKIs in 1 L, only 160 had subsequent therapy. Of 347 patients who received EGFR-TKIs in 2 L+, 134 had subsequent therapy. Most patients on subsequent therapy were confirmed to have disease progression at chart review, while the remaining were not documented to have progressed. Of patients who received EGFR-TKI in 1 L and 2 L+, 136/160 (85.0%) and 115/134 (85.8%), respectively, had disease progression.

Overall, median age at diagnosis was 69.0 years, 497 (63.6%) patients were female, 440 (56.3%) were white, 681 (87.1%) were treated in community-based practices, and 493 (63.0%) had a history of smoking (Table 3). With regards to tumor histology, most (83.8%) patients had non-SCC, 12.5% had SCC, and 3.7% had a tumor of unspecified histology. In total, 413 (52.8%) patients had a documented ECOG assessment, among whom 315 (76.3%) had an ECOG performance status of 0/1. Overall median follow-up was 309.0 days. The median time from metastatic NSCLC diagnosis until date of EGFR-TKI initiation (index date) was 62 and 328 days for patients who initiated EGFR-TKI in 1 L and 2 L+, respectively. From diagnosis to index date, 555 (71.0%) patients overall were tested for EGFR mutations. Among these patients tested, 355 (64.0%) patients were EGFR mutation-positive and, of these, five (1.4%) were T790M positive. Patients who received EGFR-TKI in 1 L were significantly older, more likely female, and less likely to be treated in community-based practices and have a history of smoking than patients who received EGFR-TKI in 2 L+. Patients who received EGFR-TKI in 1 L were significantly less likely to undergo EGFR mutation testing from diagnosis to index date compared with those who received EGFR-TKI in 2 L+ (292 [67.1%] versus 263 [75.8%], *P* = 0.008). However, among those who underwent EGFR mutation testing, patients who received EGFR-TKI in 1 L were more likely than patients who received EGFR-TKI in 2 L+ to be EGFR mutation-positive (242 [82.9%] versus 113 [43.0%], *P* < 0.001). Notably, blood-based EGFR testing was lower in patients who received EGFR-TKI in 1 L and 2 L+ (20 [6.8%] versus 40 [15.2%], *P* = 0.153).

**Table 3:** Demographic and clinical characteristics of patients who received EGFR-TKI

	Overall [A]	Patients who received 1 L EGFR-TKI [B]	Patients who received 2 L+ EGFR-TKI [C]	*P*-value for [B] vs [C]
	(*N* = 782)	(*N* = 435)	(*N* = 347)	
Observation period, days, mean ± SD [median]	371.5 ± 312.3 [309.0]	435.5 ± 329.7 [372.0]	291.3 ± 268.7 [220.0]	< 0.001
Age at metastatic NSCLC diagnosis, years, mean ± SD [median]	67.9 ± 10.2 [69.0]	69.4 ± 10.5 [70.0]	66.1 ± 9.5 [66.0]	< 0.001
Female, *n* (%)	497 (63.6)	315 (72.4)	182 (52.4)	< 0.001
Race, *n* (%)				
White	440 (56.3)	246 (56.6)	194 (55.9)	0.857
Asian	68 (8.7)	43 (9.9)	25 (7.2)	0.186
Black	73 (9.3)	40 (9.2)	33 (9.5)	0.881
Other	111 (14.2)	56 (12.9)	55 (15.9)	0.236
Unknown	90 (11.5)	50 (11.5)	40 (11.5)	0.988
Geographic region, *n* (%)				
Northeast	142 (18.2)	81 (18.6)	61 (17.6)	0.707
West	132 (16.9)	76 (17.5)	56 (16.1)	0.621
Midwest	111 (14.2)	51 (11.7)	60 (17.3)	0.027
South	284 (36.3)	145 (33.3)	139 (40.1)	0.052
Unknown	107 (13.7)	79 (18.2)	28 (8.1)	< 0.001
Community-based practice, *n* (%)	681 (87.1)	359 (82.5)	322 (92.8)	< 0.001
Insurance type, *n* (%)^a,b^				
Commercial Health Plan	379 (48.5)	198 (45.5)	181 (52.2)	0.065
Medicare	300 (38.4)	169 (38.9)	131 (37.8)	0.754
Other payer	246 (31.5)	127 (29.2)	119 (34.3)	0.127
Patient assistance program	60 (7.7)	11 (2.5)	49 (14.1)	< 0.001
Medicaid	47 (6.0)	17 (3.9)	30 (8.6)	0.006
Other government program	31 (4.0)	11 (2.5)	20 (5.8)	0.021
Self pay	7 (0.9)	4 (0.9)	3 (0.9)	0.935
Unknown	115 (14.7)	73 (16.8)	42 (12.1)	0.066
History of smoking, *n* (%)				
History of smoking	493 (63.0)	234 (53.8)	259 (74.6)	< 0.001
No history of smoking	287 (36.7)	199 (45.7)	88 (25.4)	< 0.001
Unknown	2 (0.3)	2 (0.5)	0 (0.0)	0.206
Histology at NSCLC diagnosis, *n* (%)				
Non-squamous cell carcinoma	655 (83.8)	402 (92.4)	253 (72.9)	< 0.001
Squamous cell carcinoma	98 (12.5)	19 (4.4)	79 (22.8)	< 0.001
NSCLC histology - not otherwise specified	29 (3.7)	14 (3.2)	15 (4.3)	0.417
Time from metastatic NSCLC diagnosis to index date, days, Q1 | Median | Q3				
Overall	44 | 202 | 478	22 | 62 | 336	177 | 328 | 553	< 0.001
Stage I-IIIA	29 | 158 | 505	17 | 50 | 312	171 | 372 | 589	< 0.001
Stage IIIB-IV	72 | 221 | 472	25 | 90 | 337	182 | 319 | 552	< 0.001
ECOG assessment during the baseline period (including index date), *n* (%)	413 (52.8)	212 (48.7)	201 (57.9)	0.011
ECOG performance status nearest or on the index date				
0	126 (30.5)	79 (37.3)	47 (23.4)	0.002
1	189 (45.8)	84 (39.6)	105 (52.2)	0.010
2	82 (19.9)	40 (18.9)	42 (20.9)	0.606
3	16 (3.9)	9 (4.2)	7 (3.5)	0.688
4	0 (0.0)	0 (0.0)	0 (0.0)	–
EGFR testing between the date of NSCLC diagnosis and the index date, *n* (%)				
Tested	555 (71.0)	292 (67.1)	263 (75.8)	0.008
Positive	355 (64.0)	242 (82.9)	113 (43.0)	< 0.001
T790M mutation	5 (1.4)	3 (1.2)	2 (1.8)	0.693
Exon 19 deletion	141 (39.7)	100 (41.3)	41 (36.3)	0.366
L858R point mutation in exon 21	134 (37.7)	104 (43.0)	30 (26.5)	0.003
Double mutation	9 (2.5)	5 (2.1)	4 (3.5)	0.411
Other mutation type	48 (13.5)	19 (7.9)	29 (25.7)	< 0.001
Unknown	18 (5.1)	11 (4.5)	7 (6.2)	0.509
Negative	175 (31.5)	42 (14.4)	133 (50.6)	< 0.001
Results pending	2 (0.4)	0 (0.0)	2 (0.8)	0.135
Unsuccessful/indeterminate test	23 (4.1)	8 (2.7)	15 (5.7)	0.080
Sample type among those tested				
Tissue	482 (86.8)	263 (90.1)	219 (83.3)	0.018
Blood	60 (10.8)	20 (6.8)	40 (15.2)	0.002
Unknown	14 (2.5)	10 (3.4)	4 (1.5)	0.153

*1 L* first line, *2 L+* second or later line, *ECOG* European Oncology Cooperative Group, *EGFR* epidermal growth factor receptor, *EGFR-TKI* epidermal growth factor receptor-tyrosine kinase inhibitor, *NSCLC* non-small cell lung cancer, *Q1* first quartile, *Q3* third quartile; SD: standard deviation

^a^ Evaluated on the index date

^b^ Patients may have primary and secondary insurance (i.e. patients may have more than one type of insurance). Unknown insurance type was defined as patients without any types of insurance

### EGFR mutation testing and treatment patterns

#### Patients who received subsequent therapy

**Fig. 2:**
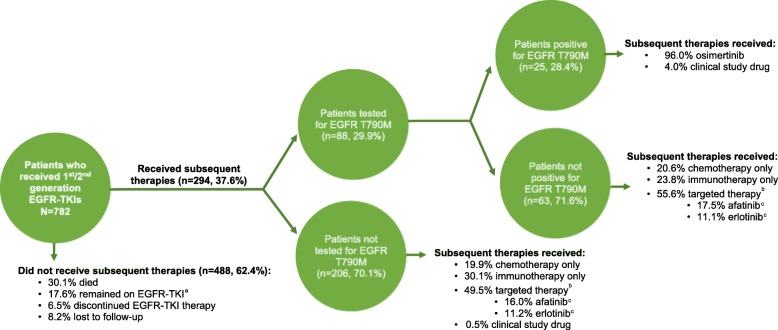
Therapies received among all patients who received EGFR-TKI and were tested for EGFR T790M. EGFR: epidermal growth factor receptor; TKI: tyrosine kinase inhibitor. ^a^ Patients still treated with their first EGFR-TKI therapy in the month prior to data cutoff were considered as remaining on EGFR-TKI therapy. ^b^ Targeted therapies included EGFR-TKIs, monoclonal antibody inhibitors, ALK inhibitors, and angiogenesis inhibitors. ^c^ The proportion of patients who received afatinib, erlotinib, and osimertinib are calculated over the total number of patients in the subgroup (e.g. the 206 patients for the analysis of patients not tested for EGFR T790M).

Of 782 patients with metastatic NSCLC who received EGFR-TKI in 1 L or 2 L+, 294 (37.6%) had subsequent therapy, while the remainder did not (235 [30.1%] died, 138 [17.6%] remained on initial EGFR-TKI, 51 [6.5%] discontinued EGFR-TKI, and 64 [8.2%] were lost to follow-up; Fig. 2).

#### Patients who received EGFR-TKI in 1 L

Of 160 (36.8%) patients who had subsequent therapy, 25 (15.6%) received chemotherapy only, 22 (13.8%) immunotherapy only, and 112 (70.0%) targeted therapies (Table 4); 99 (61.9%) patients received targeted therapies only, three (1.9%) targeted therapy plus immunotherapy, and 10 (6.2%) targeted therapy plus chemotherapy (Table 2). More specifically, 40 (25.0%) received osimertinib and 62 (38.8%) continued on or switched to another EGFR-TKI (afatinib: 33 [20.6%], erlotinib: 17 [10.6%], gefitinib: 13 [8.1%]; one patient received > 1 EGFR-TKI) as subsequent therapy. Of the 160 patients, 9 (5.6%) continued on initial EGFR-TKI plus added on chemotherapies, immunotherapies or other targeted therapies as subsequent therapy.

**Table 4:** Treatment patterns among patients with metastatic NSCLC with a subsequent therapy

Subsequent therapy	Patients who received 1 L EGFR-TKI and had subsequent therapy	Patients who received 2 L+ EGFR-TKI and had subsequent therapy
	(*N* = 160)	(*N* = 134)
Chemotherapy alone, *n* (%)	25 (15.6)	29 (21.6)
Immunotherapy alone, *n* (%)	22 (13.8)	55 (41.0)
Targeted therapies *n* (%)^a^	112 (70.0)	49 (36.6)
EGFR-TKI^b^	102 (63.8)	33 (24.6)
Continued on/switched to another 1st/2nd gen. EGFR-TKI	62 (38.8)	27 (20.1)
Afatinib	33 (20.6)	11 (8.2)
Erlotinib	17 (10.6)	13 (9.7)
Gefitinib	13 (8.1)	5 (3.7)
Osimertinib^c^	40 (25.0)	7 (5.2)
Other targeted therapies^d^	10 (6.3)	16 (11.9)
Clinical study drug, *n* (%)	1 (0.6)	1 (0.7)

*1 L* first line, *2 L+* second or later line, *ALK* anaplastic lymphoma kinase *EGFR* epidermal growth factor receptor, *EGFR-TKI* epidermal growth factor receptor-tyrosine kinase inhibitor, *NSCLC* non-small cell lung cancer

^a^ Patients could have received targeted therapies alone or in combination with chemotherapy and/or immunotherapy

^b^ Four patients had more than one EGFR-TKI as a subsequent therapy. Hence, the sum of the individual EGFR-TKIs (i.e. afatinib, erlotinib, geftinib, and osimertinib) will be greater than the total number who received EGFR-TKI

^c^ Of the 47 patients who received osimertinib as subsequent therapies, 24 were tested T790M positive, seven were not positive for T790M, and 16 were not tested for EGFR mutations

^d^ Other targeted therapeutic agents are listed in Table 1

**Fig. 3:**
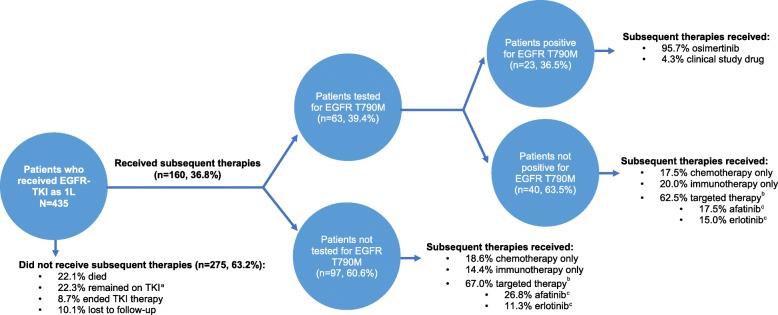
Therapies received among patients who received EGFR-TKI in first line and were tested for EGFR T790M. 1 L: first line; EGFR: epidermal growth factor receptor; TKI: tyrosine kinase inhibitor. ^a^ Patients still treated with their first EGFR-TKI therapy in the month prior to data cutoff were considered as remaining on EGFR-TKI therapy. ^b^ Targeted therapies included EGFR-TKIs, monoclonal antibody inhibitors, ALK inhibitors, and angiogenesis inhibitors. ^c^ The proportion of patients who received afatinib, erlotinib and osimertinib are calculated over the total number of patients in the subgroup (e.g. the 97 patients for the analysis of patients not tested for EGFR T790M).

Of the 160 patients, 63 (39.4%) were tested for T790M between index date and start of subsequent therapy (Fig. 3). Among the 63 patients, 23 (36.5%) were T790M positive; of these, 22 (95.7%) received osimertinib and one (4.3%) received a clinical study drug. Among the 40 patients who were T790M negative, seven (17.5%) received chemotherapy only, eight (20.0%) immunotherapy only, and 25 (62.5%) targeted therapies (afatinib: seven [17.5%]; erlotinib: six [15.0%]; osimertinib: five [12.5%]; gefitinib: three [7.5%]; other: four [10.0%]). In the remaining 97 (60.6%) patients not tested for EGFR mutations in the post-EGFR-TKI treatment period, 18 (18.6%) received chemotherapy only, 14 (14.4%) immunotherapy only, 65 (67.0%) targeted therapies (afatinib: 26 [26.8%]; erlotinib: 11 [11.3%]; osimertinib: 13 [13.4%]; gefitinib: 10 [10.3%]; other: six [6.2%]).

#### Patients who received EGFR-TKI in 2 L+

Of the 134 (38.6%) patients with subsequent therapy, 29 (21.6%) received chemotherapy only, 55 (41.0%) immunotherapy (Table 4); 27 (20.1%) received targeted therapies only, two (1.5%) targeted therapy plus immunotherapy, and 20 (14.9%) targeted therapy plus chemotherapy (Table 2). Specifically, seven (5.2%) received osimertinib, and 27 (20.1%) continued or switched to another EGFR-TKI (afatinib: 11 [8.2%]; erlotinib: 13 [9.7%]; gefitinib: five [3.7%]) as subsequent therapy. Of the 134 patients, nine (6.7%) continued on initial EGFR-TKI plus added on chemotherapies, immunotherapies or other targeted therapies as subsequent therapy.

**Fig. 4:**
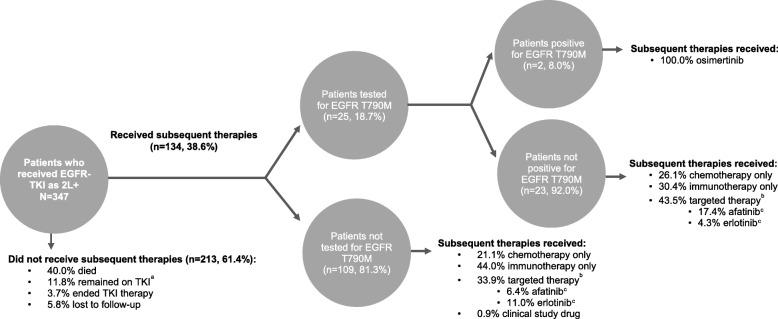
Therapies received among patients who received EGFR-TKI in second or later line and were tested for EGFR T790M. 2 L+: second or later line; EGFR: epidermal growth factor receptor; TKI: tyrosine kinase inhibitor. ^a^ Patients still treated with their first EGFR-TKI therapy in the month prior to data cutoff were considered as remaining on EGFR-TKI therapy. ^b^ Targeted therapies included EGFR-TKIs, monoclonal antibody inhibitors, ALK inhibitors, and angiogenesis inhibitors. ^c^ The proportion of patients who received afatinib, erlotinib and osimertinib are calculated over the total number of patients in the subgroup (e.g. the 109 patients for the analysis of patients not tested for EGFR T790M).

Among the 134 patients, 25 (18.7%) were tested for T790M (Fig. 4). Among the 25 patients tested, two (8.0%) were T790M positive and both received osimertinib. Among the 23 T790M negative patients, six (26.1%) received chemotherapy only, seven (30.4%) immunotherapy only, and 10 (43.5%) targeted therapies (afatinib: four [17.4%]; osimertinib: two [8.7%]; erlotinib: one [4.3%]; other: three [13.0%]). In the remaining 109 patients not tested for T790M in the post-EGFR-TKI treatment period, 23 (21.1%) received chemotherapy only, 48 (44.0%) immunotherapy only, and 37 (33.9%) targeted therapies (erlotinib: 12 [11.0%]; afatinib: seven [6.4%]; gefitinib: five [4.6%]; osimertinib: three [2.8%]; other: 13 [11.9%]).

Additional details on EGFR mutation testing between index date and subsequent therapy, and results are presented in Table 5.

**Table 5:** EGFR mutation testing between index date and subsequent therapy

Types of Biomarkers	Patients with subsequent therapy	Patients who received 1 L EGFR-TKI and had subsequent therapy	Patients who received 2 L+ EGFR-TKI and had subsequent therapy
	(***N*** = 294), *n* (%)	(***N*** = 160), *n* (%)	(***N*** = 134), *n* (%)
Tested	88 (29.9)	63 (39.4)	25 (18.7)
Positive^a^	47 (53.4)	39 (61.9)	8 (32.0)
T790M mutation	25 (53.2)	23 (59.0)	2 (25.0)
Exon 19 deletion	21 (44.7)	16 (41.0)	5 (62.5)
L858R point mutation in exon 21	11 (23.4)	9 (23.1)	2 (25.0)
Other mutation type	2 (4.3)	1 (2.6)	1 (12.5)
Unknown	0 (0.0)	0 (0.0)	0 (0.0)
Negative	38 (43.2)	22 (34.9)	16 (64.0)
Results pending	2 (2.3)	1 (1.6)	1 (4.0)
Unsuccessful / indeterminate test	1 (1.1)	1 (1.6)	0 (0.0)
Sample			
Tissue	48 (54.5)	36 (57.1)	12 (48.0)
Blood	35 (39.8)	24 (38.1)	11 (44.0)
Unknown	3 (3.4)	2 (3.2)	1 (4.0)
Urine	2 (2.3)	1 (1.6)	1 (4.0)

*1 L* first line, *2 L+* second or later line, *EGFR* epidermal growth factor receptor, *EGFR-TKI* epidermal growth factor receptor-tyrosine kinase inhibitor

^a^ EGFR mutations are reported among those patients with a positive EGFR test result. Patients may have more than one EGFR mutation

## Discussion

This study provided a novel opportunity to document treatment patterns, use of EGFR mutation testing, and how patients with metastatic NSCLC are managed in a real-world clinical setting based on data from EHR used by US-based cancer care providers. These data illuminated several notable gaps in care. Despite clinical treatment guidelines recommending routine EGFR testing for all patients with metastatic NSCLC of non-SCC histology [6–8], here only 71% of newly diagnosed patients underwent EGFR testing prior to EGFR-TKI initiation. Consistent with this observation, another observational research study showed rates of documented EGFR testing rate to range between 41 and 97% in patients with newly-diagnosed metastatic NSCLC initiating systemic therapy (including EGFR-TKIs) [20]. Furthermore, the 30% rate of EGFR testing before subsequent therapy is consistent with previous estimates (30–48%) [21–23] and emphasizes that, during the study period, a substantial proportion of patients initiated a subsequent therapy without updated information on the molecular profile of the tumor.

In this study, 87% of patients were treated in the community setting. Community-based practices may not be implementing standard testing guidelines or may have barriers related to EGFR mutation testing. Prior research has highlighted logistical challenges for community-based oncologists regarding mutation testing, such as coordination of sample handling, long turnaround times, and test reimbursements, which may affect adherence to testing guidelines during the treatment process for patients with NSCLC [24]. Lastly, other barriers in all settings include inability to wait for testing and delay start of treatment, tissue insufficiency, potential harm from repeat biopsies, poor patient health and inability to undergo testing [24, 25]. Of note, 12.5% of included patients had SCC, and these patients are less likely to have undergone testing due to the low frequency of EGFR mutations and differences in clinical guidelines [6–8].

Our study reported median time from metastatic NSCLC diagnosis to EGFR-TKI initiation in 1 L of 62 days. A recent scoping review reported mean/median time from NSCLC diagnosis to treatment ranging from 15 to 60 days in the US [26]. There are limited studies evaluating time from metastatic NSCLC diagnosis to treatment; however, one study reported 29% of patients waited more than 90 days from initial presentation to clinician to treatment, of whom 49% were stage IIIA-IV [27]. Prior studies have suggested challenges to achieving shorter time to treatment, including delayed return of results and lack of communication of positive findings to the clinician and/or patient, while some are administrative including changes in insurance for patients [26, 27]. This underscores the need for broad-based education throughout the oncology community, plus further research to understand EGFR mutation testing uptake and barriers to accessing current state technologies.

Since 2013, clinical guidelines recommend repeat molecular testing upon progression on first- and second-generation EGFR-TKIs to detect mutations such as T790M [7]. Prior to 2015, there were no standard therapeutic options for patients with metastatic NSCLC with acquired resistance to initial EGFR-TKI [28]. Osimertinib has led to a paradigm shift in the management of patients with metastatic NSCLC who develop T790M and experience disease progression on first- or second-generation EGFR-TKIs. The need for tumor genotyping is thus highly supported at least at time of diagnosis and disease progression on or after first- or second-generation EGFR-TKIs for mutation evaluation. Nevertheless, only approximately 30% of patients in this study who received first- or second-generation EGFR-TKIs and progressed were tested for T790M prior to initiating subsequent therapy. The proportion of patients tested for EGFR mutations on progression prior to initiating subsequent therapy was lower in patients treated with an EGFR-TKI in 1 L versus 2 L+ (39% versus 19%). Among patients tested for T790M, 28% were positive and mostly received osimertinib. One rationale for the lower proportion of patients tested for EGFR mutations on progression may be that EGFR testing was not recommended prior to FDA approval of osimertinib [6–8]; therefore, EGFR testing following progression was slowly being adopted during this study. In any case, 30% of patients who received first- or second-generation EGFR-TKI died before receiving any subsequent therapy and only 38% of patients treated with EGFR-TKI had subsequent therapy. Another recent study reported that 30% of patients with EGFR mutations were not treated with subsequent therapy due to fast disease progression and death [29].

In previous studies among patients with NSCLC who progressed on 1 L EGFR-TKI therapy, approximately 50% were T790M positive [10, 11]. Our study indicates a lower proportion of T790M positive patients (1 L = 37%, 2 L+ =8%). This lower rate is at least partially due to the fact that almost half of patients included in our study lacked evidence of EGFR-TKI sensitizing mutations at baseline. Lower rates of T790M detection may also be attributed to use of plasma/serum (blood) for EGFR testing, and sensitivities of laboratory techniques in the detection of the mutation [30, 31]. Recent prospective studies showed higher T790M detection rates in tissue versus plasma samples [30, 32]. In our study, either tissue (1 L = 57%; 2 L+ =38%) or blood (1 L = 48%; 2 L+ =44%) was used for EGFR testing upon progression. Depending on the laboratory technique used to detect T790M, sensitivities can range from as low as 29–82%, and specificities from 83 to 100% [31]. Laboratory techniques with lower sensitivities may not accurately identify T790M. The database does not detail methods of detection (e.g. Cobas [Roche], ddPCR [Bio-Rad]) used to identify T790M; therefore, we cannot be certain that observed lower T790M detection rates are attributable to detection methods associated with low sensitivities.

In April 2018, osimertinib received approval as 1 L treatment in patients with EGFR ex19del/L858R-positive metastatic NSCLC [33]. The approval was based on the FLAURA trial which demonstrated that, in previously-untreated EGFR mutation-positive NSCLC, median progression-free survival (osimertinib = 18.9 months, comparator = 10.2 months, hazard ratio = 0.46) and overall survival (osimertinib = 38.6 months, comparator = 31.8 months, hazard ratio = 0.80) were longer among patients receiving osimertinib compared with standard EGFR-TKIs [4, 16]. More recently, a global multicenter retrospective (GioTag) study demonstrated a median time on chemotherapy-free treatment of 27.6 months among patients with metastatic NSCLC with EGFR- mutation (ex19del/L858R) who received 1 L afatinib followed by second-line osimertinib [34]. Although there was clinical benefit in sequential treatment of afatinib followed by osimertinib, findings from the current study show that 30% of the patients who received first- or second-generation EGFR-TKI died before receiving any subsequent therapy, and only 30% were tested for T790M upon progression, resulting in a small number of patients (47 [16%]) actually receiving osimertinib as a subsequent therapy option. Consequently, many patients with metastatic NSCLC may not have survived long enough to be EGFR-tested and treated with osimertinib as subsequent therapy. The low rate of documented EGFR mutation positivity at baseline may have contributed to this high mortality rate, since EGFR-TKI therapy may have suboptimal efficacy in this setting. Given the results from FLAURA, considering 1 L treatment that could maximize clinical benefit for all eligible EGFR mutation-positive patients will be an important treatment decision for patients moving forward. Since there has been evolution in the treatment landscape of EGFR mutation-positive patients with approval of 1 L osimertinib, future research is needed to evaluate its impact on treatment patterns, outcomes in the real-world setting, and the molecular basis of acquired resistance to osimertinib.

This study has several strengths. First, with a nationally representative dataset including both structured and unstructured data processing, the database offers a unique opportunity to study disease progression among cancer patients. Second, the large patient population was drawn primarily from community-based practices (> 85%); results of this study are generalizable to US community-based oncology practices [35]. Third, slightly more patients who were T790M negative received immunotherapy alone (1 L = 20%) vs chemotherapy alone (1 L = 18%) as subsequent therapy, despite clinical guidelines recommending chemotherapy in the second-line setting for T790M negative patients [6]. These unique findings provide invaluable insight to real-world community practice patterns. Lastly, we evaluated testing and treatment patterns in the overall study sample, and further stratified patients who received EGFR-TKI in 1 L and 2 L+, enabling assessment of characteristics of testing and treatment patterns by line of therapy.

Some limitations of the study should be noted. First, data on some clinically important patient characteristics were not available in the database. For example, ECOG performance status was available for approximately 50% of patients, therefore description of patient characteristics is based on available data. Second, the indication for erlotinib changed in late 2016 for patients with NSCLC receiving maintenance or 2 L+ treatment, to limit use to those whose tumors have EGFR ex19del or L858R mutations [36]. Therefore, some patients in the current study who were not EGFR mutation-positive may have received erlotinib as second-line or maintenance therapy. The subset of patients who received EGFR-TKI in 1 L may thus be more reflective of real-world mutation testing and treatment practices. Lastly, since data are drawn from community oncology centers, results may not be representative of practice at US academic medical centers.

## Conclusions

This study provides insight into mutation testing and treatment patterns in patients with metastatic NSCLC in real-world, US, community-based practices. Findings showed that, in the community-based setting, approximately 30% of patients were tested for T790M following treatment with first- or second-generation EGFR-TKI. Among patients who underwent EGFR-TKI resistance testing, the most common EGFR mutation was T790M. Patients were initiated on 1 L EGFR-TKI therapy on average 2 months after the initial diagnosis. These significant delays signal the presence of medical and administrative barriers that hinder the rapid initiation of EGFR-TKI therapy; overcoming these barriers may be key to optimize patient outcomes. Given that 30% of the patients died without receiving any further therapy and that only 30% of those who progressed were tested for T790M, this study highlights the importance of choosing the most optimal treatment option for patients with EGFR-mutated NSCLC in the 1 L setting.

## Data Availability

Data used for this study are from Flatiron Health and were used under license for the current study. Therefore, restrictions apply to the availability of these data, which are not publicly available. Access to this data set is available to other interested parties for a fee set by Flatiron Health (https://flatiron.com/contact/). No administrative permissions were required to access the raw data.

## Abbreviations

1 L: first line
2L+: second or later line
EGFR: Epidermal growth factor receptor
EHR: Electronic health records
ex19del: exon 19 deletion
NSCLC: Non-small cell lung cancer
TKI: Tyrosine kinase inhibitor
